# Impact of Co-Infections and BCG Immunisation on Immune Responses among Household Contacts of Tuberculosis Patients in a Ugandan Cohort

**DOI:** 10.1371/journal.pone.0111517

**Published:** 2014-11-05

**Authors:** Irene A. Biraro, Moses Egesa, Frederic Toulza, Jonathan Levin, Stephen Cose, Moses Joloba, Steven Smith, Hazel M. Dockrell, Achilles Katamba, Alison M. Elliott

**Affiliations:** 1 College of Health Sciences, Makerere University, Kampala, Uganda; 2 Medical Research Council/Uganda Virus Research Institute, Uganda Research Unit on AIDS, Entebbe, Uganda; 3 Department of Immunology and Infection, London School of Hygiene and Tropical Medicine, London, United Kingdom; 4 Department of Clinical Research, London School of Hygiene and Tropical Medicine, London, United Kingdom; National Institute for Infectious Diseases (L. Spallanzani), Italy

## Abstract

**Background:**

Tuberculosis incidence in resource poor countries remains high. We hypothesized that immune modulating co-infections such as helminths, malaria, and HIV increase susceptibility to latent tuberculosis infection (LTBI), thereby contributing to maintaining the tuberculosis epidemic.

**Methods:**

Adults with sputum-positive tuberculosis (index cases) and their eligible household contacts (HHCs) were recruited to a cohort study between May 2011 and January 2012. HHCs were investigated for helminths, malaria, and HIV at enrolment. HHCs were tested using the QuantiFERON-TB Gold In-Tube (QFN) assay at enrolment and six months later. Overnight whole blood culture supernatants from baseline QFN assays were analyzed for cytokine responses using an 11-plex Luminex assay. Associations between outcomes (LTBI or cytokine responses) and exposures (co-infections and other risk factors) were examined using multivariable logistic and linear regression models.

**Results:**

We enrolled 101 index cases and 291 HHCs. Among HHCs, baseline prevalence of helminths was 9% (25/291), malaria 16% (47/291), HIV 6% (16/291), and LTBI 65% (179/277). Adjusting for other risk factors and household clustering, there was no association between LTBI and any co-infection at baseline or at six months: adjusted odds ratio (95% confidence interval (CI); p-value) at baseline for any helminth, 1.01 (0.39–2.66; 0.96); hookworm, 2.81 (0.56–14.14; 0.20); malaria, 1.06 (0.48–2.35; 0.87); HIV, 0.74 (0.22–2.47; 0.63). HHCs with LTBI had elevated cytokine responses to tuberculosis antigens but co-infections had little effect on cytokine responses. Exploring other risk factors, Th1 cytokines among LTBI-positive HHCs with BCG scars were greatly reduced compared to those without scars: (adjusted geometric mean ratio) IFNγ 0.20 (0.09–0.42), <0.0001; IL-2 0.34 (0.20–0.59), <0.0001; and TNFα 0.36 (0.16–0.79), 0.01.

**Conclusions:**

We found no evidence that co-infections increase the risk of LTBI, or influence the cytokine response profile among those with LTBI. Prior BCG exposure may reduce Th1 cytokine responses in LTBI.

## Introduction

Tuberculosis (TB) is a complex disease with a global burden of 8.7million (8.3–9.0million) incident cases and an annual mortality of approximately 9million deaths. In 2011, Uganda had an estimated annual TB incidence of 234 per 100 000 population and it ranked 19^th^ on the list of top 22 TB endemic countries [1]. The World Health Organization (WHO) estimates that one third of the world population is infected with latent tuberculosis (LTBI) [2], and may thus act as a reservoir for active TB disease. In addition to a high TB burden, Uganda also has a high prevalence of malaria (45%) [3], adult HIV (7.2%) [4], and many neglected tropical diseases such as soil transmitted helminths including hookworm (54%), *Trichuris trichiura* (5%), *Ascaris lumbricoides* (6.3%), and Schistosomiasis (0.9%) [5].

In the recent past, there has been a particular interest in whether infections endemic to the tropics modulate host immunity, resulting in increased susceptibility to TB and thereby maintaining the TB epidemic, especially in low income countries such as Uganda [6], [7]. Some of these co-infections have been well studied, in particular the role of HIV in fueling the spread of tuberculosis [8]–[10], but the relationship between TB and other co-infections such as helminths, malaria and cytomegalovirus (CMV) remains to be clearly documented.

Protection against intracellular organisms such as *Mycobacterium tuberculosis* (M.tb) is mainly cell mediated and particularly involves T-helper (Th) 1-type cytokines (interferon (IFN) γ, tumour necrosis factor (TNF) α, and interleukin-2 (IL-2)), and to some extent Th17 cytokines [11]. Helminths have been shown to modulate immunity toward Th2 and regulatory responses, and away from Th1-type responses, and this may make individuals infected with helminths more susceptible to M.tb infection [12]–[17].

During the early erythrocytic stage of malaria infection, there is an increased production of Th1-type cytokines, which not only promotes parasite elimination, but also immunopathology, leading to the symptoms of malaria [18]–[20]. However, malaria infection is also associated with depression of adaptive immune responses, allowing parasites to evade elimination [21]–[23] and making individuals with malaria infection potentially susceptible to infections normally controlled by cell mediated immunity such as *Herpes simplex* reactivation [24] and, we postulated, M.tb infection.

Cytomegalovirus infection, which is endemic in developing countries [25] infects children *in-utero*, and early in life, causing clinical or sub-clinical disease and thereafter establishing latency [26], [27]. This common viral infection is associated with dysfunction and terminal differentiation of T-cells [28]–[30] and could potentially diminish the protective responses against TB.

We carried out a longitudinal observational study to determine the association between helminths, malaria, CMV, or HIV and LTBI among household contacts exposed to sputum positive TB with the hypothesis that individuals who are infected with these pathogens, and are at the same time exposed to persons with active TB, would be particularly susceptible to acquiring M.tb infection.

## Methods

### Study design and setting

This was a cohort study with six months of follow up. Participants were recruited at Kitebi and Kisenyi health centers in Kampala city, Uganda from May 2011 to January 2012. The city is divided into five divisions each with a serving health center. Kitebi health center is located in the peri-urban Rubaga division while Kisenyi health center is found in Kampala central division which is a densely populated slum. Both health centers run out-patient tuberculosis clinics twice a week and offer tuberculosis testing and treatment.

### Study participants

Newly diagnosed sputum smear positive patients above 18 years of age attending the clinics were screened for eligibility to participate in the study as index cases. Index cases starting or who had been on TB therapy for one month or less, with household contacts (HHCs), were consecutively included.

All available members of the index case's household were screened. Members that had stayed and shared meals with the index case for more than two weeks before the index case was diagnosed with TB irrespective of age and gender were approached to participate in the study.

The HHCs were divided into three major groups depending on their LTBI status and all were followed up for six months. Those that were HIV positive or below 5years of age received six months of isoniazid preventive therapy (IPT) according to the WHO guidelines [31]. The HHCs that were HIV negative and above 5 years of age with LTBI were randomly assigned to two groups, one receiving IPT and monthly visits, and the other receiving only monthly visits.

### Data collection

Questionnaires were administered both to the index cases and to HHCs. Data on socio-demographics, household characteristics, TB related risk factors and exposures, medical history, and clinical findings were collected. Both index cases and HHCs received HIV counseling and testing, and they provided a sample of 3 mls of blood for the QuantiFERON-TB Gold In-Tube (Cellestis GmbH (Europe), Hannover, Germany) test (QFN). Cut off for a positive QFN test is >0.35 IU/ml after subtracting the nil (unstimulated) IFNγ production from production in the antigen-stimulated tube, and >0.5 IU/ml after subtracting nil from production in the mitogen-stimulated tube [32]. In addition, HHCs provided 1 ml of blood for malaria parasitology (assessed by thick film and Giemsa stain), and *Mansonella perstans* parasitology (assessed by Knott's method [33] and Giemsa stain) and 1 ml for CMV serology (Diasorin S.P.A., Saluggia, Italy). HHCs were also asked to give three consecutive daily stool samples. These were analyzed using the Kato-Katz method [34] at the Vector Control Division of the Ministry of Health, Uganda.

Two hundred and twenty samples of QFN supernatants from the household contacts were analyzed for cytokine responses using an 11-analyte Bio-plex Pro cytokine assay (Bio-Rad, Richmond, USA) (Luminex) consisting of Th1 cytokines (detection limits) (IFNγ (92.6–52,719 pg/ml), IL-2 (2.1–17,772 pg/ml), TNFα(5.8–95,484 pg/ml)), Th2 cytokines (IL-4 (2.2–3,467 pg/ml), IL-5 (3.1–7,380 pg/ml), IL-13 (3.7–3,137 pg/ml)), regulatory cytokine (IL-10 (2.2–8,840 pg/ml)), and Th17 cytokines (IL-17a (4.9–12,235 pg/ml), IL-17f (3.04–18,668 pg/ml), IL-21 (8.97–147,023 pg/ml), IL-22 (3.88–11,917 pg/ml)). These cytokines were measured from the nil (no antigen) and TB antigens (ESAT-6, CFP-10 and TB7.7 (peptide 4)) tubes for each HHC.

### Statistical analysis

A sample size of 145 index cases was chosen. We assumed an average of two HHCs per index case; a prevalence of 10% for malaria, helminth, and HIV infections and 70% for CMV infection; a prevalence of 30% for LTBI in the HHCs without any co-infections; a design effect due to clustering within the households of 1.2; and a loss to follow up of 9%. This sample size would give 80% power to detect as statistically significant increase at the 5% level in LTBI from 30% to 60% among the HHCs with any co-infection using the formula of Fleiss JL *et al.,* [35] without a continuity correction.

The main outcome of this study was infection with latent tuberculosis defined as a positive result to the QFN test at baseline. Similar associations were explored for LTBI at the end of follow up, and for an additional outcome, QFN conversion between baseline and follow up. The four main exposure variables were co-infection with helminths, malaria, HIV and CMV.

Other exposure factors assessed were characteristics of the household (type of house, sanitation, exposure and ventilation factors, water sources, type of fuel used for cooking, lighting, and distance to the health centers), of the index case (age, gender, educational level, ability to read and write, occupation and income, religion, tribe, marital status, number of children, number of household members, HIV status, duration of cough, and sputum positivity levels), and of the HHCs (sex, age, socio-economic status, relationship to index case, daily social interaction or proximity with index case, daily contact time with index patient, smoking, alcohol use, presence of BCG scar). These were selected based on prior social and biological plausibility [36], [37]. The socio-economic status score was based on the type of roof and walls of the house, and lighting used. We scored the ventilation based on number of windows and doors that were opened daily to the outside per room. Interaction with the index case was categorized as: (i) only shared meals with the index case, (ii) cared for and shared meals with the index case but did not share the same room, (iii) shared the same bedroom but not the same bed (iv) shared the same bedroom and bed.

First unadjusted associations with LTBI were assessed by using cross tabulations and by fitting logistic regression models that took account of the clustering of household contacts using a robust approach. The Wald test was used to assess statistical significance. Then, a multivariable analysis was conducted using a stepwise backward elimination approach to exclude potential confounders, using a liberal p-value of 0.15 to ensure that no important confounder was omitted [38].

The Luminex data obtained was adjusted to exclude individual analyte values generated with bead counts of less than 20 as follows: Values above 1 pg/ml but below the lower detection limit (DL) for each cytokine were set to half the lower DL for that cytokine. Values above the upper DL were set to the upper DL. Negative values and values between 0 and 1 pg/ml were set to 1 pg/ml. Since the cytokine responses were skewed, the data were subjected to a logarithmic (to base 10) transformation.

Graphs were generated to show cytokine distributions among the groups of interest in the spontaneous (no antigen) and stimulated (TB antigen) data. Despite the log transformation, the data on cytokine responses remained non-normal with a heavy-tailed distribution. We therefore fitted linear regression models to the log-transformed cytokine responses, but used bootstrap resampling methods to estimate the standard errors, in order to investigate associations between the cytokine responses and exposures to different co-infections and other risk factors [39].

### Ethical approval

The study was approved by both the Makerere University Ethical Review Board and Uganda National Council of Science and Technology. All the Index cases gave written informed consent to participate in the study, including permission allowing the team to visit their homes. All adult HHCs gave written informed consent to participate in the study. Parents, next of kin, caretakers or/guardians gave written informed consent on behalf of the minors or children to participate in the study. In addition, children between 10 and 17 years also gave written informed assent to participate in the study.

The HHCs who were infected with helminths or malaria were treated with anti-helminthic or anti-malaria drugs, respectively at the health center, as promptly as possible.

## Results

**Figure 1:**
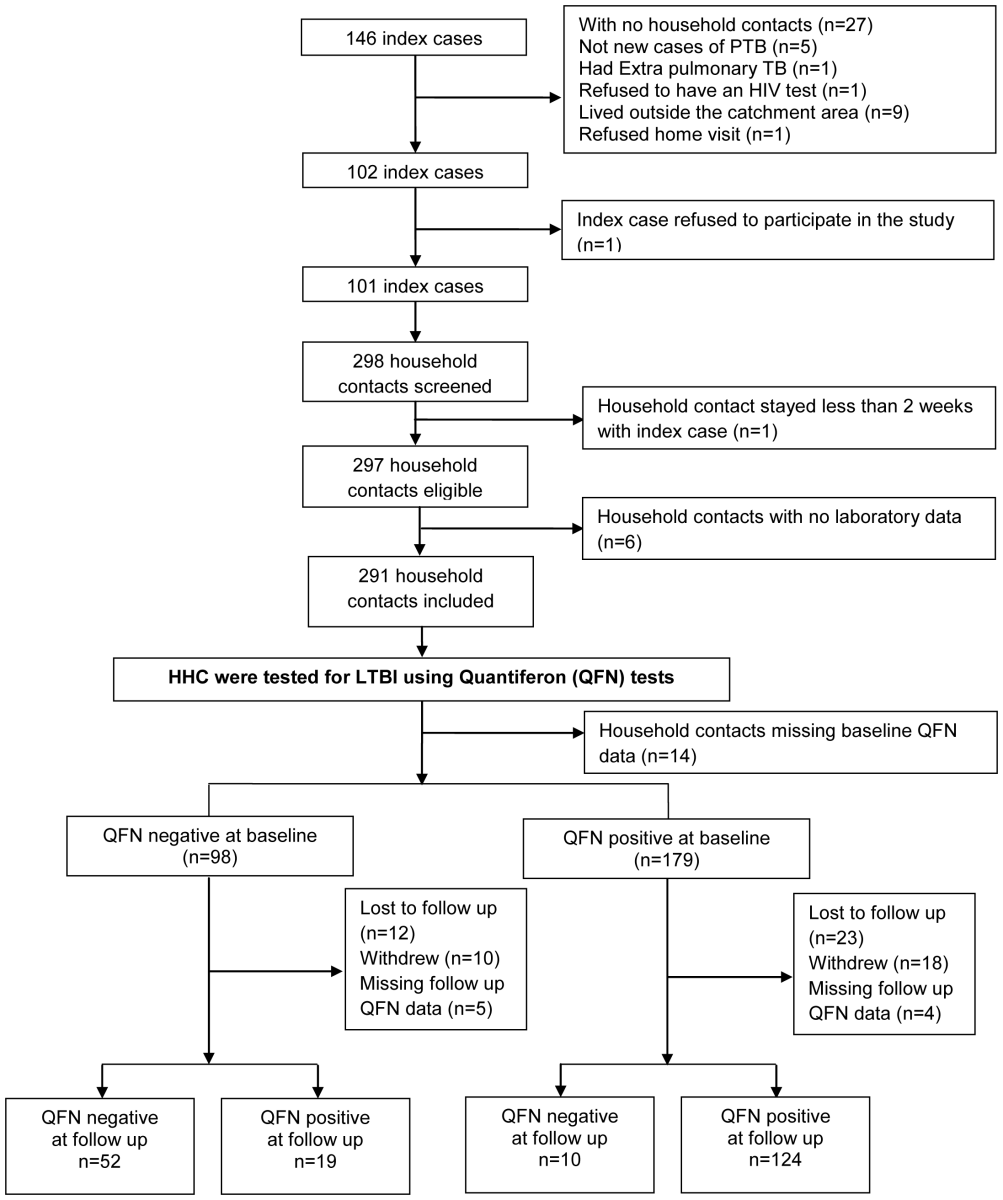
Flow diagram showing the recruitment process.

The study recruited 101 index cases and 291 HHCs who were followed up as detailed in figure 1.

### Participant characteristics

The median age (interquartile range (IQR)) of the index cases was 28 years (23–35 years) and 57% (58/101) were male. Regarding employment, 38% (38/101) were unskilled labourers, 6% (6/101) skilled, 28% (29/101) self-employed and 28% (28/101) lacked employment.

The median age (IQR) of the HHCs was 14 years (5–26 years) and 42% (123/291) were male.

### Household characteristics

We assessed 101 households of which 60% (61/101) were one roomed houses. The median number of household members (IQR) was 3 (2–4). Fifty six percent (56/101) of the houses had poor ventilation, 34% (34/101) average ventilation, and 10% (10/101) good ventilation. Over 96% (96/101) used charcoal or firewood as a fuel for cooking. Regarding sanitation, 92% (93/101) had access to a pit latrine. These were being shared by a median (IQR) of 10 (6–15) people. Eighty three percent (84/101) had access to clean water from communal taps. It took an average (SD) of 38 (18) minutes for the household members to get to the study health centers with equal numbers using various transport means which included walking to the health centers (32%), using public motorcycles called boda bodas (32%), and using the public minibus taxis (34%).

### Prevalence of co-infections and LTBI among HHCs

Among HHCs, the prevalence of helminths was 9% (25/291), malaria 16% (47/291), and HIV 6% (16/291). Of the 16 who were HIV positive, 3 were of known status and were on antiretroviral therapy (zidovudine, lamivudine and nevirapine). Four HHC had both helminths and malaria, 5 HHC had malaria and HIV, and no HHC had all the three infections.

Fifty nine HHCs provided all the three samples of stool required, 71 provided 2, and 115 provided only one sample. An individual was categorized as helminth infected if any species was detected in at least one of all stool samples available for each HHC. Results were hookworm (13 cases), *Trichuris trichiura* (5 cases), *Hymenolepis nana* (3 cases), *Schistosoma mansoni* (3 cases), and *Ascaris lumbricoides* (1 case). There were two HHCs with dual helminth infections; one had both hookworm and *Ascaris lumbricoides*, and another had *Hymenolepis nana* and *Schistosoma mansoni*. None of the HHCs had *Mansonella perstans*. Over 97% of the HHCs were infected with CMV and therefore associations between this infection and LTBI could not be investigated in the analysis. The prevalence of LTBI (QFN positive) was 65% (179/277), and the rest of the analysis was based on this definition.

There were 45 HHCs with missing information on helminth infections, and 21 HHCs with missing information on malaria status. We assessed whether there was any relationship between the missingness and our main outcome (LTBI) by testing for any association between the missing data and LTBI using a Chi-square test. We found no significant difference between the patients with complete and missing data.

### Epidemiological factors associated with LTBI

In the univariable analysis, co-infection with helminths, malaria or HIV was not associated with increased risk of LTBI (Table 1) but factors such as age of the HHC, proximity of the HHC to the index case, HHCs who smoked or took alcohol were strongly associated with LTBI. The crude odds ratio for the association between co-infection with hookworm, which was the most common helminth, and LTBI was large (3.85) in line with our hypothesis. However due to the small number of participants with helminth infection the confidence interval was wide and the association did not reach statistical significance (P = 0.09).

**Table 1:** Univariable analysis of risk factors associated with LTBI among household contacts.

Risk Factors	Level	QFN Negative (n = 98) n (%)	QFN Positive (n = 179) n (%)	Crude OR (95% CI)	P-values
**Factors relating to the household contact**					
Helminths	No	81 (39)	129 (61)	1	0.32
*45 missing	Yes	7 (29)	17 (71)	1.52 (0.65–3.54)	
Hookworm	No	86 (39)	134 (61)	1	0.09
*45 missing	Yes	2 (14)	12 (86)	3.85 (0.79–18.68)	
Malaria	No	73 (34)	143 (66)	1	0.51
*21 missing	Yes	18 (39)	28 (61)	0.79 (0.39–1.58)	
HIV	No	91 (35)	170 (65)	1	0.50
	Yes	7 (44)	9 (56)	0.68 (0.22–2.08)	
Sex	Female	56 (35)	103 (65)	1	0.95
	Male	42 (36)	76 (64)	0.98 (0.58–1.66)	
Age	(median (IQR))	10 (4–20)	20 (9–30)	1.02 (1.00–1.05)	0.01
Social-economic Status	Lower	47 (36)	83 (64)	1	0.88
*5 missing	Higher	50 (35)	92 (65)	1.04 (0.58–1.85)	
Relationship to index case	First degree relative	55 (31)	123 (69)	1	0.08
	Not first degree relative	43 (43)	56 (57)	0.58 (0.31–1.08)	
Proximity to index case	Shared meals with index case	31 (61)	20 (39)	1	<0.0001
*3 missing	Cared for index case	17 (25)	52 (75)	4.74 (2.23–10.04)	
	Shared room with index case	39 (37)	65 (63)	2.58 (1.21–5.48)	
	Shared bed with index case	10 (20)	40 (80)	6.20 (2.67–14.34)	
Duration of contact with index case	>6hrs/day	79 (34)	154 (66)	1	0.23
	<6hrs/day	19 (43)	25 (57)	0.67 (0.35–1.29)	
Smoking	No	94 (39)	145 (61)	1	0.0007
	Yes	4 (11)	34 (89)	5.51 (2.05–14.78)	
Alcohol	No	88 (40)	134 (60)	1	0.002
*2 missing	Yes	10 (19)	43 (81)	2.82 (1.44–5.50)	
BCG scar	No	18 (30)	42 (70)	1	0.32
*2 missing	Yes	80 (37)	135 (63)	0.72 (0.37–1.38)	
Factors relating to the Index case					
Index case HIV positive	Negative	67 (32)	140 (68)	1	0.09
*14 missing	Positive	27 (48)	29 (52)	0.51 (0.23–1.12)	
Sputum positivity	1+	33 (46)	39 (54)	1	0.11
*8 missing	2+	24 (29)	60 (71)	2.11 (1.00–4.46)	
	3+	38 (34)	75 (66)	1.67 (0.86–3.20)	
Index case cough duration (months)*7 missing	Median (IQR)	2 (1–5)		1.05 (0.93–1.17)	0.38

Relationship to Index: ^#^First degree  =  Spouse, Son/Daughter, Father, Mother, Brother, Sister, Step-mother, Step-Father, Step-child, Step-sister, Step-brother. ^##^Not first degree  =  Cousin, Niece/Nephew, Aunt/Uncle, Grandparent, Grandchild, other relative, and non-related

After adjusting for confounders, factors which remained associated with risk of LTBI were closer proximity of HHC to the index case, and HHCs that smoked. HHCs were less likely to have acquired LTBI if their index case was HIV positive (Table 2).

**Table 2:** Multivariate analysis of risk factors associated with LTBI among household contacts at baseline.

Risk Factor	Level	Adjusted OR (95% CI)	P-value
Helminths	No	1	0.96
	Yes	1.01 (0.39–2.66)	
Hookworm	No	1	0.20
	Yes	2.81 (0.56–14.14)	
Malaria	No	1	0.87
	Yes	1.06 (0.48–2.35)	
HIV	No	1	0.63
	Yes	0.74 (0.22–2.47)	
Proximity to index case	Shared meals with index case	1	<0.0001
	Cared for index case	4.99 (2.19–11.39)	
	Shared room with index case	3.53 (1.61–7.77)	
	Shared bed with index case	10.57 (4.19–26.65)	
Smoking	No	1	0.001
	Yes	7.68 (2.32–25.47)	
Index case HIV positive	Negative	1	0.02
	Positive	0.32 (0.12–0.83)	

Immunosuppressive co-infections might impair the response in the QFN assay, as well as increasing susceptibility to LTBI and this might mask a true positive association between co-infection and LTBI. Treatment of co-infection with malaria or helminths was possible and we postulated that, if such masking had occurred, treatment might reveal a previously hidden association between co-infection during the period of exposure to the active case and LTBI. Therefore, having treated helminth and malaria co-infections at baseline, we investigated for associations between baseline co-infections and LTBI status at the end of six months of follow up. None of the co-infections was associated with LTBI status at 6 months (Table 3).

**Table 3:** Multivariate analysis of risk factors associated with LTBI among household contacts at end of follow up.

Risk Factor	Level	Adjusted OR (95% CI)	P-value
Helminths	No	1	0.26
	Yes	0.51 (0.16–1.65)	
Hookworm	No	1	0.67
	Yes	0.69 (0.12–3.81)	
Malaria	No	1	0.09
	Yes	0.49 (0.22–1.12)	
HIV	No	1	0.90
	Yes	0.86 (0.07–9.41)	
Age group (years)	0–5	1	0.00001
	6–12	1.53 (0.41–5.68)	
	13–18	2.90 (0.59–14.10)	
	>18	12.21 (3.83–38.93)	
Proximity to index case	Shared meals with index case	1	0.00001
	Cared for index case	3.96 (1.34–11.66)	
	Shared room with index case	9.61 (3.53–26.10)	
	Shared bed with index case	33.61 (6.19–182.55)	
Index case HIV positive	Negative	1	0.011
	Positive	0.24 (0.08–0.72)	

Some of the HHC, particularly the older ones, might have been exposed to, and infected with, M.tb before contact with this particular index case, in which case current co-infections, even if chronic, might not be relevant to their LTBI status at baseline. Therefore, the relationship between co-infections at baseline and QFN conversions during the six months of follow up were explored among 19 QFN converters and 52 individuals who remained QFN negative. Small numbers gave this analysis limited power, but there was no suggestion of an association with co-infection (aOR (95% CI) for helminths 0.29 (0.02–3.00), for malaria 0.17 (0.02–1.16) and for HIV 0.24 (0.003–19.2). In this model only male gender aOR 3.44 (95% CI 1.15–10.32) and daily duration of time with the index case greater than six hours 5.42 (1.38–21.3) were associated with QFN conversion.

### Cytokine responses in HHCs

**Figure 2:**
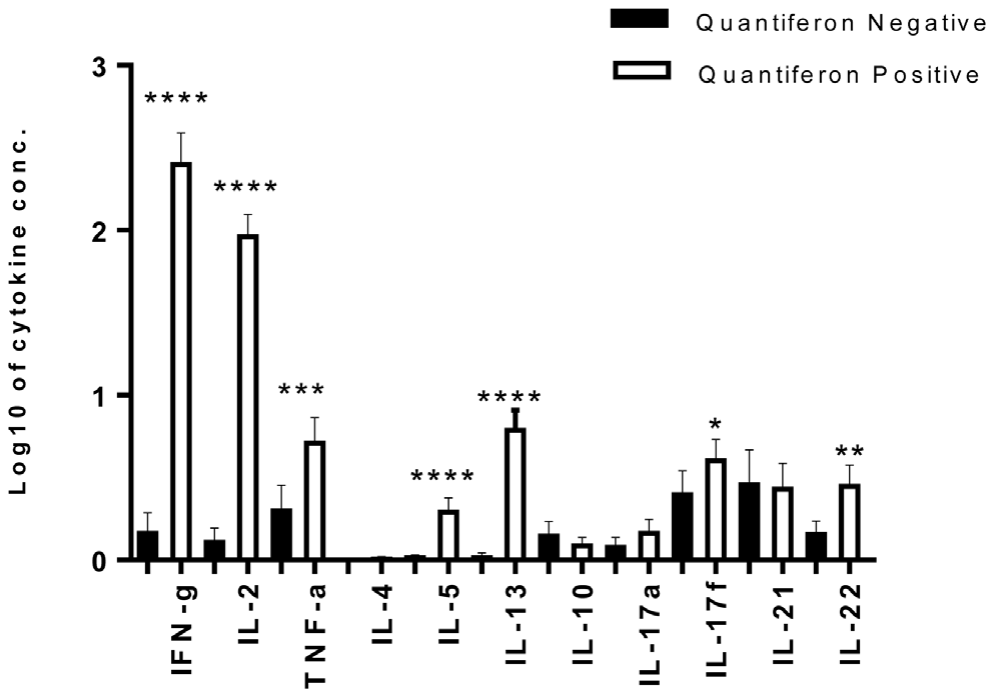
Cytokine responses in HHCs who were Quantiferon (QFN) negative (LTBI negative) or QFN positive (LTBI positive). Means with 95% CI of net cytokine production were determined in response to TB antigens (ESAT-6, CFP-10 and TB7.7 (peptide 4)) in QFN plasma supernatants tested for cytokine content using multiplex bead array. Spontaneous cytokine production was subtracted prior to analysis. The t-test was used to compare means between QFN negative and QFN positive individuals. **** p<0.0001; *** p<0.001; ** p<0.01; * p = 0.05.

Control responses measured in the mitogen tube were high for all HHCs. Spontaneous cytokine production (measured in the no antigen tube) varied between cytokines, from geometric mean 1.11 pg/ml for IL-4 to 67.35 pg/ml for IFNγ, but did not differ between HHCs with or without LTBI, except for IFNγ (geometric mean 71.96 and 57.83 pg/ml, respectively, p = 0.04). Spontaneous production was subtracted from the amount produced in the TB antigen stimulated tube for further analysis. By definition, HHCs with LTBI had a higher IFNγ response to TB antigens than uninfected contacts in the QFN assay. IFNγ responses to TB antigens measured by Luminex correlated strongly with IFNγ measured in the QFN assay (Pearson's correlation coefficient, r = 0.83, p = <0.0001) and so, as expected, IFNγ responses to the TB antigens measured by Luminex were higher in the HHCs with LTBI compared to those without LTBI (p = <0.0001) (Figure 2). Similarly, many of other cytokine responses to TB antigens were also higher in the HHCs with LTBI contacts: Th1 cytokines IL-2 (p = <0.0001), TNFα (p = 0.0009); Th2 cytokines IL-5 (p = <0.0001), IL-13 (p = <0.0001); Th17 cytokines IL-17f (p = 0.05), IL-22 (p = 0.002). However there was no difference between HHCs with or without LTBI in the concentrations of the regulatory cytokine IL-10, or of the Th17 cytokines IL-17a and IL-21 in the TB-antigen-stimulated QFN supernatants.

### Effect of co-infections on cytokine responses among HHCs

Spontaneous cytokine production was similar among household contacts with and without co-infections except for those with malaria who had raised IL-17f (geometric mean 32.53 vs 14.12 pg/ml, respectively, p = 0.006) and IL-21 (geometric mean 50.66 vs 22.50 pg/ml, respectively, p = 0.03) and those with HIV who had depressed concentrations of IL-10 (geometric mean 2.03 vs 4.81 pg/ml, respectively, p = 0.01).

Co-infections and other factors could only be expected to influence cytokine responses to TB-antigens among individuals with LTBI, so further analysis was restricted to this subset.

**Figure 3:**
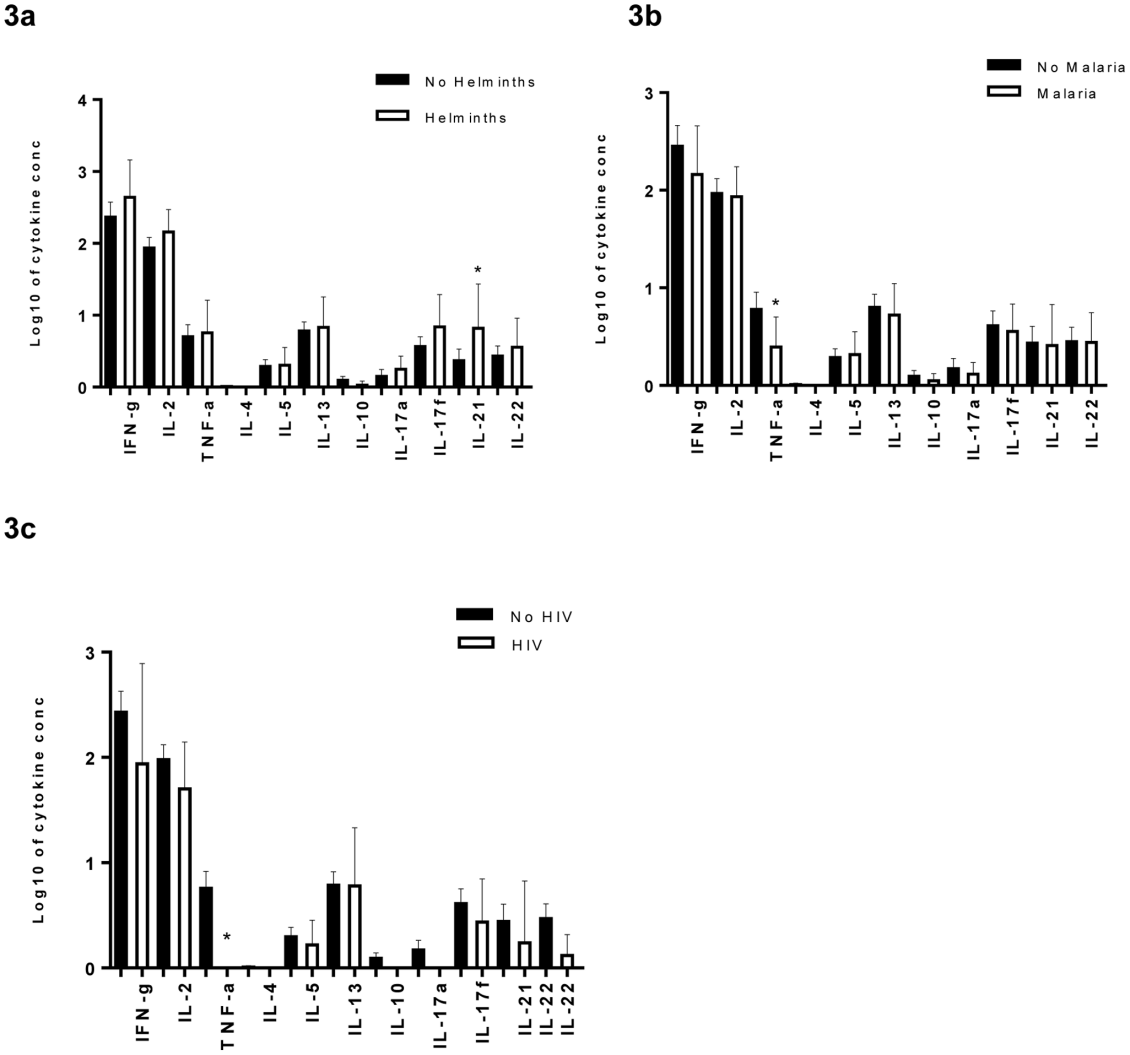
Cytokine responses among HHCs with LTBI. We assessed the effect of the three co-infections on the cytokine responses of the HHCs with LTBI: A) helminths, B) malaria, and C) HIV. Means with 95% CI were determined and the t-test was used to compare HHCs who were co-infected (bold bars) or not (clear bars). * p<0.01.

In general, co-infections showed little effect on net TB-antigen stimulated cytokine responses, except for increased IL-21 production in HHCs with helminths compared to those without helminths (geometric mean 6.07 vs 2.45 pg/ml, respectively, p = 0.05), decreased TNFα levels in malaria versus no malaria (geometric mean 1.83 vs 4.36 pg/ml, respectively, p = 0.05), and reduced TNFα production in HIV versus no HIV co-infection (geometric mean 1.00 vs 4.04 pg/ml, respectively, p = 0.01)(figures 3a, 3b, 3c).

We performed crude and adjusted analyses of the different cytokine responses among the HHCs with LTBI and did not find any association between the helminth co-infections and cytokine responses to TB antigens (table 4) but confirmed that malaria co-infection was associated with reduced levels of TNFα (aGMR 0.39 (0.18–0.85)), and HIV co-infection with reduced cytokine production of TNFα (aGMR 0.19 (0.09–0.39)), IL-10 (aGMR 0.77(0.65–0.91)), IL-17a (aGMR 0.68(0.53–0.87)), and IL-22 (aGMR 0.44 (0.26–0.74)).

**Table 4:** Multivariable analysis of factors associated with cytokine responses among household contacts that had LTBI.

Cytokine Group	Risk Factor	Adjusted GMR (95% CI)	P-value
**Pro-inflammatory cytokines**			
**IFN-γ**	BCG scar	0.20 (0.09–0.42)	<0.0001
**IL-2**	BCG scar	0.34 (0.20–0.59)	<0.0001
**TNF-α**	BCG scar	0.36 (0.16–0.79)	0.01
	Co-infection with malaria	0.39 (0.18–0.85)	0.01
	Co-infection with HIV	0.19 (0.09–0.39)	<0.0001
**Th2 cytokines**			
**IL-5**	Higher socio-economic status	0.54 (0.31–0.96)	0.03
	Sputum positivity		
	2+	1.42 (0.81–2.50)	0.03
	3+	1.64 (1.12–2.40)	
	Index case HIV positive	0.46 (0.26–0.81)	0.007
**IL-13**	Higher socio-economic status	0.36 (0.180.72)	0.004
	Sputum positivity		
	2+	1.89 (0.85–4.21)	0.07
	3+	2.04 (1.06–3.94)	
**Regulatory cytokines**			
**IL-10**	BCG scar	1.23 (1.04–1.45)	0.01
	Spending <6hr/day with index case	0.76 (0.64–0.90)	0.001
	Co-infection with HIV	0.77 (0.65–0.91)	0.002
**Th17 cytokines**			
**IL-17a**	Co-infection with HIV	0.68 (0.53–0.87)	0.003
**IL-22**	Co-infection with HIV	0.44 (0.26–0.74)	0.002

Geometric mean ratios with 95% CI are shown from linear regression models with bootstrapping to demonstrate associations between individual cytokine response outcomes and exposure to the different co-infections and other risk factors among household contacts with LTBI.

### Other factors associated with cytokine responses and LTBI

The multivariable analyses revealed other factors of interest that were associated with cytokine responses among LTBI infected HHCs. The presence of a BCG scar was associated with markedly low concentrations of all the Th1 cytokines IFNγ, IL-2, and TNFα (aGMR 0.20 (0.09–0.42), 0.34 (0.20–0.59), and 0.36 (0.16–0.79) respectively) but raised IL-10 levels (aGMR 1.23 (1.04–1.45)).

Reduced Th2 cytokines were associated with household contacts with higher social-economic status (IL-5: aGMR 0.54(0.31–0.96), IL-13: 0.36(0.18–0.72)). These same cytokines, in the LTBI positive contacts, were also increased with increasing sputum positivity in the index case.

## Discussion

In our study, co-infection with helminths, malaria or HIV was not associated with increased risk of LTBI. In keeping with previous studies, the risk of LTBI increased with closer intensity of exposure with the index case, with those HHCs who shared the same bedroom and bed with the index cases, or spent many hours per day with the index case, being at the most risk [40], [41]. Our findings also further support the hypothesis that smoking increases the risk of LTBI. The underlying mechanism which makes smokers more susceptible to TB is believed to be related to the depression of adaptive immune responses by tobacco [42]–[44]. The household contacts of HIV positive index cases had a reduced risk of LTBI, in keeping with previous studies that have shown that HIV positive TB cases are less infectious than those who are HIV negative [45]–[47].

The HHCs with LTBI had elevated cytokine responses to *M.* tb-specific antigens although we did not show any specific Th-type bias. Previous studies have found distinct Th1- type responses in LTBI compared to cases with TB disease [48], [49]. A study by Mahan *et al*., showed higher concentrations of IFNγ responses in contacts with LTBI compared to those without LTBI [50]. Here, they focused on IFNγ responses and they diagnosed LTBI using the tuberculin skin test while we assessed a range of immune responses and used the QFN test. We also found strong Th1- type responses but in addition, the other groups of effector cytokines were raised in people with LTBI.

When we assessed the effect of co-infections on cytokine responses in the HHCs who had LTBI, we found that malaria and HIV showed reduced cytokine responses across all cytokine families, with statistically significant associations for TNFα, in keeping with their known immune suppressive effects. However, helminth co-infection was not associated with any reduction in cytokine response. The reduced cytokine responses to TB antigens in HIV co-infection, and in particular IFNγ responses, concurs with previous studies [51], [52], and might be expected to result in increased susceptibility to TB infection and disease as has been reported before [53]–[56]. It was therefore surprising that we saw no evidence of an overall increase in LTBI in the HHCs with HIV, however our analysis was limited by lack of data on CD4 counts and hence on the level of immunosuppression in these contacts.

We also found that having a BCG scar was associated with low Th1-type but high IL-10 (regulatory) cytokine responses among the HHCs with LTBI. This was an unexpected finding, and could have arisen by chance, but the consistency of the results between the three Th1 cytokines lends weight to the observation. Other studies have also shown a negative association between BCG scar and interferon gamma release assay (IGRAs) positivity, which has been taken to imply protection against LTBI [57]–[59]. Although our study showed no evidence of overall protection, based on the QFN result, one possible explanation of the lower Th1-type cytokine concentrations is that BCG conferred sufficient protection to reduce the acquired bacterial load [60], [61]. About 45% of the household contacts were below 12 years of age and might still be in the age bracket to benefit from protection of their BCG vaccination [62], [63].

By contrast, a household contact study by Whalen *et al*., carried out in a different part of Kampala city, found that whereas BCG reduced the risk of tuberculosis infection as was shown by the reduced risk of TST conversions, the household contacts with a BCG scar who acquired LTBI (TST converters) had elevated IFNγ responses to mycobacterial antigen at baseline, compared to TST converters who did not have a BCG scar. However, Whalen and colleagues used *M. tuberculosis* culture filtrates for stimulation and the tuberculin skin test to diagnose LTBI [64], and so their findings may have been influenced by exposure to non-tuberculous mycobacteria, perhaps boosting a BCG-primed response, while our results are presumed to reflect exposure and susceptibility specifically to *M. tuberculosis* infection. The observed high concentrations of IL-10 found in those with a BCG scar are consistent with findings which show that BCG induced macrophage or dendritic cell dependent production of IL-10 may prevent T-cell induced immunopathology [65].

The prevalence of helminths and malaria was low and may have been underestimated, since about half the participants provided only a single sample [66]. In the case of helminths, we attribute the low prevalence to the aggressive treatment of helminthiasis by the Ministry of Health of Uganda following the WHO recommendations [67], [68] to actively deworm school going children and the general population seeking care at peripheral health centers, including those where the study was carried out. However only 6% (16/290) of the household contacts in this study reported that they had received any form of anti-helminth treatment in the past month. Thus, although we achieved the required sample size, the power of the study was lower than expected to detect associations between LTBI and individual helminth species, and it is plausible that effects of different species may differ. We did not to include Strongyloides among the helminths we investigated for because it is not detected using the Kato Katz method which we were using. This species is generally less commonly detected in our environment than the other species, so it is unlikely that we missed many cases [69].

In addition, the study used the QFN test for the diagnosis of LTBI. This test assumes that the body is able to mount an immune response against the TB antigens resulting in production of IFNγ. The co-infections investigated are associated with immune suppression, in particular HIV and malaria. Infection with HIV has a definite effect on the performance of the tuberculin skin test when used to diagnose LTBI [70], [71] although the effect on QFN is less clear, with some associating the test with indeterminate results or low sensitivity of diagnosing LTBI [72]–[74], and others showing good results [75], [76].

It is plausible that the household contacts with these co-infections were unable to produce adequate amounts of IFNγ making it difficult to see an association with LTBI. This was addressed for helminths and malaria by repeat QFN testing after treatment of the co-infections, and the results did not change. Detailed information on antiretroviral therapy uptake in the HHCs with HIV infection was not available, but there was still no association between HIV and LTBI among the contacts at the end of follow up.

## Conclusions

We found no evidence that the co-infections in question (helminths, malaria, and HIV) increase the risk of latent TB acquisition. We have also shown that BCG could be modulating the Th1 and regulatory immune response profile in LTBI. It is important to further explore the effect of BCG on potential clinical outcomes.
